# Engineering microbial cell factories for the production of plant natural products: from design principles to industrial-scale production

**DOI:** 10.1186/s12934-017-0732-7

**Published:** 2017-07-19

**Authors:** Xiaonan Liu, Wentao Ding, Huifeng Jiang

**Affiliations:** 1 Key Laboratory of Systems Microbial Biotechnology, Tianjin Institute of Industrial Biotechnology, Chinese Academy of Sciences, Tianjin, China; 2 University of Chinese Academy of Sciences, Beijing, China

**Keywords:** Plant natural products, Synthetic biology, Microbial cell factories, Metabolic engineering, Industrial production

## Abstract

Plant natural products (PNPs) are widely used as pharmaceuticals, nutraceuticals, seasonings, pigments, etc., with a huge commercial value on the global market. However, most of these PNPs are still being extracted from plants. A resource-conserving and environment-friendly synthesis route for PNPs that utilizes microbial cell factories has attracted increasing attention since the 1940s. However, at the present only a handful of PNPs are being produced by microbial cell factories at an industrial scale, and there are still many challenges in their large-scale application. One of the challenges is that most biosynthetic pathways of PNPs are still unknown, which largely limits the number of candidate PNPs for heterologous microbial production. Another challenge is that the metabolic fluxes toward the target products in microbial hosts are often hindered by poor precursor supply, low catalytic activity of enzymes and obstructed product transport. Consequently, despite intensive studies on the metabolic engineering of microbial hosts, the fermentation costs of most heterologously produced PNPs are still too high for industrial-scale production. In this paper, we review several aspects of PNP production in microbial cell factories, including important design principles and recent progress in pathway mining and metabolic engineering. In addition, implemented cases of industrial-scale production of PNPs in microbial cell factories are also highlighted.

## Background

Thousands of plant natural products (PNPs) can be utilized as drugs, cosmetics, dyes, seasonings, nutraceuticals, and industrial chemicals, all of which play important roles in human life [1]. Unfortunately, in recent years over-exploitation has endangered more than 15,000 medicinal plant species in their natural habitats, which resulted in a precarious and unsustainable supply of the invaluable natural products [2, 3]. At the same time, seasonal, climatic or other environmental variations can also threaten the supply of these natural products [4]. The yield of natural products from plants also cannot satisfy the demands of the growing market. Due to the structural complexity of most natural products, total chemical synthesis approaches are often inefficient and accompanied by large amounts of waste and heavy pollution [5]. Therefore, engineering microbial cell factories to produce these high-value PNPs has become a promising solution to protect endangered plants and prevent pollution from chemical synthesis [4, 6]. Current developments in biological “omics” techniques and synthetic biology provide excellent tools to remove obstacles in the construction of microbial cell factories to produce PNPs [5, 7, 8]. Over the past few decades, there has been great progress in multiple aspects of heterologous synthesis of PNPs, including the identification of biosynthetic pathways, the construction of microbial cell factories, and the development of industrial production [9]. Engineered microbial cell factories have been successfully applied to produce PNPs from renewable carbon sources at an industrial scale. Further progress in the field of microbial cell factories will not only potentially save endangered plants, but also profoundly change the traditional ways of PNP production.

**Fig. 1:**
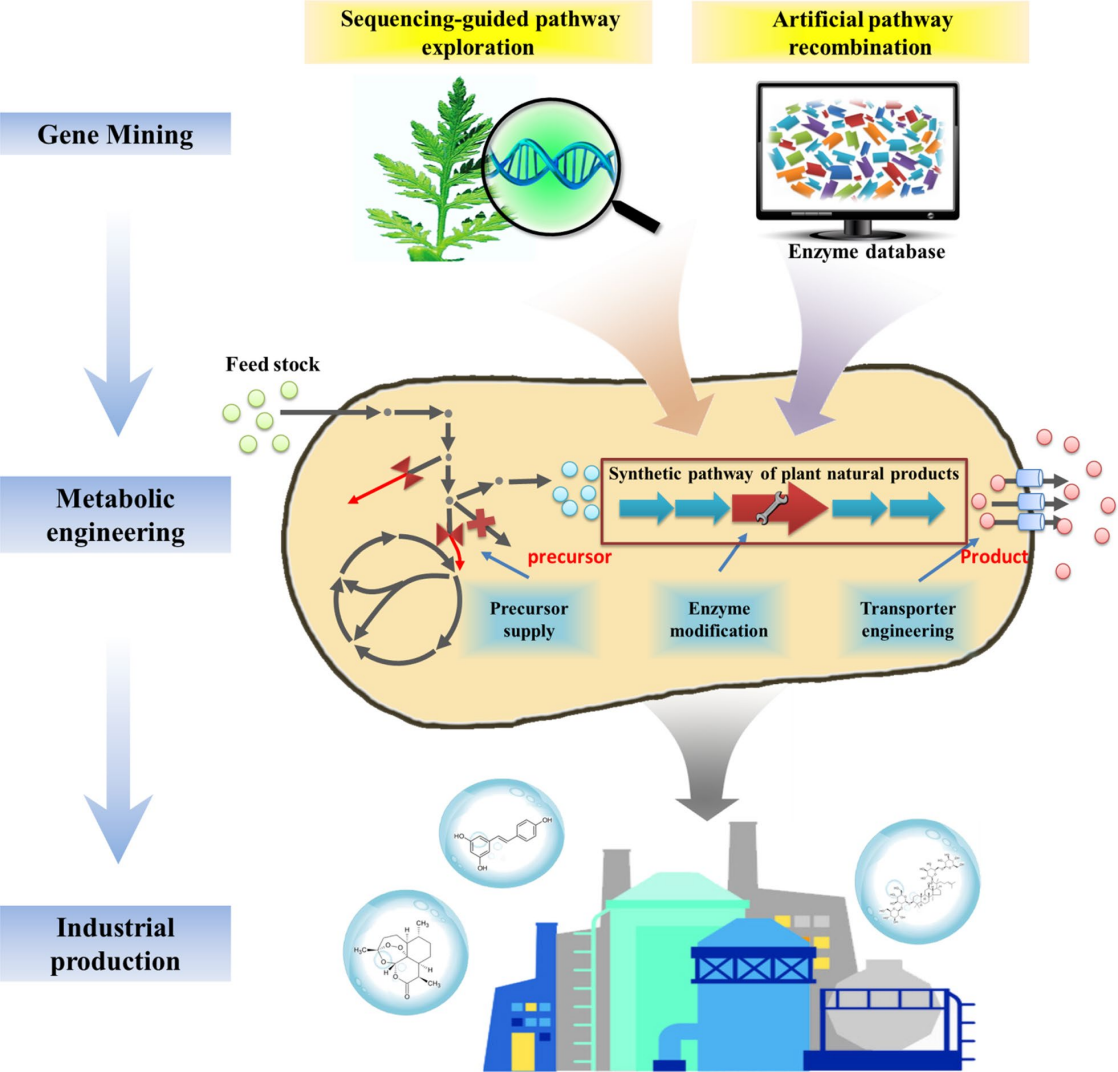
A schematic summary of microbial cell factories design, optimization and industrial production. The mining of biosynthetic pathways of plant natural products by sequencing-guided pathway exploration and artificial pathway recombination. Optimization of microbial cell factories by improving precursor supply, enzyme modification and transporter engineering. Industrial-scale production of three natural products (artemisinin, resveratrol and carotenoids) is shown as an example

In this review, we summarize the recent progress of microbial cell factory engineering, identification of novel biosynthetic pathways, and the production of PNPs on an industrial scale. Firstly, two tentative strategies based on biological “omics” and synthetic biology technologies for gene mining from the biosynthetic pathways of PNPs are introduced, followed by a discussion of three pivotal steps in the optimization of microbial cell factories, including the improvement of precursor supply, enzyme activity and product transport. Finally, several milestone examples of industrial PNP production in microbial cell factories are showcased (Fig. 1).

## Mining of PNP biosynthetic pathways

The first step in the construction of a PNP-producing microbial cell factory is to identify the original biosynthetic pathway. The rapid development of molecular biology, “omics” technologies and bioinformatics, has enabled great breakthroughs in the identification of PNP-biosynthetic pathways. In this section, recent approaches in gene exploration for the production of natural compounds are reviewed.

### Sequencing-guided pathway exploration

Traditionally, reverse transcription-polymerase chain reaction (RT-PCR), rapid amplification of cDNA ends (RACE) [10–12], RNA interference (RNAi) [13, 14], virus-induced gene silencing (VIGS) [15] and isotopic tracer methods [16] had been used to uncover novel genes in the biosynthetic pathways of PNPs. However, these technologies are usually time-consuming, laborious, and consequently expensive. With the rapid development of sequencing technology, “omics” analysis and high-throughput screening technologies, thousands of functional genes related to PNP-biosynthetic pathways have been identified in recent years [17]. For example, the assembly of the *Salvia miltiorrhiza* transcriptome provided a valuable resource for the investigation of the complete biosynthetic pathway of tanshinone [18]. High-density genetic linkage mapping using recombinant inbred lines (RILs) had been used to decipher the genetic networks underlying flavonoid biosynthesis in *Brassica napus* [19]. Genes related to cucurbitacins biosynthesis were characterized by genome-wide association analysis based on the genomic variation map of 115 diverse cucumber lines [20, 21]. Whole-genome sequencing of *Siraitia grosvenorii*, combined with transcriptomic (RNA-Seq) and bioinformatic analyses made a great contribution to illuminate the biosynthetic pathway of mogroside V [22]. Glycosyltransferases of ginsenosides were also successfully cloned based on the ESTs (Expressed Sequence Tags) and cDNA database of *Panax ginseng* [23, 24]. Six enzymes acquired by RNA-Seq from mayapple were assembled into the complete biosynthetic pathway of the etoposide aglycone [25]. Therefore, “omics” technologies have greatly accelerated the pace of identification of novel genes from the biosynthetic pathways of PNPs.

### Recombined artificial biosynthetic pathways

Currently, various online databases providing enormous amounts of genomic data also facilitate the identification of novel genes. For example, the 1000 plants project (1KP) proposed to collect the transcriptomes of 1000 plant species [26]. The Medicinal Plant Genomics Resource provides transcriptome and metabolome resources from medicinal plants [27]. Phytozome is a comparative platform for green plant genomics, providing a view of the evolutionary history of each plant’s genes, as well as access to the sequences and functional annotations [28]. Moreover, based on detailed information about genes, enzymes, reactions and their regulation, comprehensive metabolic networks of PNPs have been composed based on reactions mined from different species using bioinformatics databases such as KEGG (Kyoto Encyclopedia of Genes and Genomes) [29]. From these databases, it is therefore possible to “dig up” genes from different species to construct artificial biosynthetic pathways of PNPs for which the natural pathway is unknown. For instance, the biosynthesis of opioids in yeast is considered to be one of the most remarkable landmarks of synthetic biology, representing a highly sophisticated feat of engineering a very complex metabolic pathway in microbe [30–32]. So far, 21 enzymes were successfully reconstructed in yeast for the heterologous biosynthesis of opioids by combining the building blocks derived from multiple species, such as *Papaver somniferum, Papaver bracteatum, Coptis japonica, Eschscholzia californica, Rattus norvegicus*, *Pseudomonas putida*, and yeast [31]. Additionally, raspberry ketone [33], salidroside [34], gastrodin [35], and salvianic acid A (SAA) [36] were also successfully synthesized in microorganisms using recombined artificial pathways. Therefore, it has become feasible to construct the biosynthetic pathways of PNPs without genetic information from the original plant. Unprecedented microbial synthesis of PNPs via recombined artificial pathways provides a new perspective in the construction of biosynthetic pathways for even the most complex PNPs. With increasing information on functional genes and enzymes, recombined artificial pathways will shed light on ways to produce new PNPs in cell factories.

## Optimization of microbial cell factories

Although many biosynthetic pathways of PNPs have been identified, producing PNPs in microbial cell factories is still a tough challenge for a number of reasons. First, most PNPs are secondary metabolites, which receive relatively low levels of carbon metabolic flux in comparison with primary metabolites. Thus, it is necessary to rewire the metabolic fluxes toward their precursors in order to increase the production of PNPs. Second, due to the complexity of the molecular structures of most high-value PNPs, multiple genes are involved in the biosynthesis of these compounds. Consequently, improving the catalytic activities of so many genes in the synthetic pathway is another bottleneck. Last but not least, when the microbial host is producing PNPs, the accumulation of precursors or products in vivo can cause feedback inhibition or toxicity. In order to rescue cells from metabolite accumulation and improve the production of heterologous PNPs, transporter engineering strategies have been implemented to secrete the target metabolites outside of the cells. In this section, recent progresses in metabolic network rewiring, as well as engineering of enzymes and PNP transporters are summarized.

### Improving precursor supply

The limited supply of precursors is one of the main challenges for heterologous PNP synthesis in microbial hosts. One typical case is the heterologous production of terpenoids. The carbon skeletons of all terpene molecules are composed of the five-carbon precursors isopentenyl pyrophosphate (IPP) and dimethylallyl pyrophosphate (DMAPP), which are synthesized through the MEP pathway (2-C-methyl-d-erythritol 4-phosphate pathway) or MVA pathway (mevalonate pathway) [37]. However, the flux toward the MEP or MVA pathway is considered to be limited in terpenoid production in microbial hosts. Overexpression of a truncated *HMG1* (*tHMG1*), which catalyzes the rate-limiting step in the MVA pathway, can be used to avoid accumulating toxic amounts of *β*-hydroxy-*β*-methylglutaryl-CoA (HMG-CoA), and increase the supply of IPP or DMAPP in *Saccharomyces cerevisiae* [38]. Overexpression of all genes from the MVA pathway in *S. cerevisiae* using the GAL promoter also increased the metabolic flux [39]. Moreover, decreasing or eliminating the fluxes going toward competitive pathways is also a conventional strategy for pathway optimization. For example, to improve the production of artemisinic acid, an inducible promoter was used to reduce the expression of the *ERG9* gene and resulted in a decrease in the metabolic flux from IPP to ergosterol, which in turn pushed the conversion of IPP toward artemisinic acid [40]. Furthermore, a sequential control strategy of the precursor farnesyl pyrophosphate (FPP) has been developed in *S. cerevisiae*, which improved the production of *β*-carotenoids [41]. This offers a practical and cost-efficient approach to improve the biosynthetic production of natural compounds.

Flavonoids are widespread PNPs with high pharmaceutical value, but the production of flavonoids in recombinant microbes is low. Tyrosine or phenylalanine and malonyl-CoA are the main precursors in flavonoid biosynthesis. Introducing a tyrosine insensitive 3-deoxy-d-arabinose-heptulosonate-7-phosphate synthase mutant (encoded by *ARO4* ^*G226S*^), knocking-out *ARO3*, and phenylpyruvate decarboxylase genes (*PDC1*, *PDC5* and *PDC6*), together with overexpression of chalcone synthase (CHS) and tyrosine ammonia lyase (TAL) resulted in a 40-fold increase of extracellular naringenin titer in glucose-grown shake-flask cultures [42, 43]. In *Escherichia coli*, the acetyl-CoA carboxylase complex (*accABCD*), biotin ligase (*BirA*) from *Photorhabdus luminescens*, and enzymes in the acetate assimilation pathway [acetate kinase A (*ackA)*, phosphate acetyltransferase (*pta*) and acetyl-CoA synthase (*acs*)] were overexpressed, which largely increased flavonoid production [44]. Additionally, overexpression of *β*-ketoacyl-ACP synthase II (*FabF*) was able to increase cellular malonyl-CoA levels and pinocembrin production [45]. In recent years, a number of groups have developed strategies for dynamic regulation, which usually depend on appropriate biosensors. These strategies allow the rebalancing of fluxes according to changing conditions in the cell or the fermentation medium [46]. For example, a hybrid *cis*- and *trans*- regulatory promoter from *Bacillus subtilis*, which responds to a broad concentration range of malonyl-CoA during metabolic processes, was introduced into *E. coli* and led to the dynamic control of malonyl-CoA-associated fluxes [47]. Therefore, various metabolic engineering strategies for the regulation of precursor supply have enabled improvements in yields and titers of a variety of natural products produced in microorganisms.

### Enzyme modification

The production of metabolites is often impeded by insufficient catalytic activity of enzymes. Enzyme engineering thus is a key solution for the improvement of metabolic fluxes towards PNPs. Two main strategies have been used in enzyme engineering for higher catalytic activities. One is based on directed evolution, utilizing methods such as error-prone PCR (Polymerase Chain Reaction), random mutations and site-specific mutations. The other is based on semi-rational or rational design.

Directed evolution is a widely used strategy for altering the catalytic characteristics of enzymes. For example, the directed evolution of lycopene cyclase (CrtYB) was employed to inactivate the lycopene cyclase function but retain the phytoene synthase function for improving lycopene production in engineered *S. cerevisiae*. The catalytic activity of geranylgeranyl diphosphate synthase (CrtE) was also improved by directed evolution in order to enhance the synthesis of geranylgeranyl pyrophosphate (GGPP), and reached a production of 1.61 g/L lycopene in the engineered diploid strain by fed-batch fermentation [48]. Bai et al. reported that the catalytic properties of a glycosyltransferase UGT73B6 toward phenolic alcohol were improved through directed evolution, and the resulting strain produced much higher yield of gastrodin, reaching 545 mg/L in 48 h [35]. Through in vivo evolution of stilbene synthase, pinosylvin production was increased up to 23-fold when cerulenin was added [49]. Despite the heavy workload and low efficiency compared to rational design methods, directed evolution is still a simple and accessible method for enzyme modification, especially for those proteins whose structure is unknown.

Rational design methods are usually based on the knowledge of protein structure and catalytic mechanism. Keasling’s team engineered P450BM3 (a substrate-promiscuous P450 enzyme) from *Bacillus megaterium* via a ROSETTA-based energy minimization method, which enabled the P450BM3 mutants to conduct selective oxidation of amorphadiene, and produced artemisinic-11S,12-epoxide at titers greater than 250 mg/L in *E. coli* [50]. Ajikumar’s team modified the N-terminus of CYP725A4 and achieved the highest titer of oxygenated taxanes so far (570 ± 45 mg/L) in *E. coli* [51]. Liu et al. reported that the catalytic activity of isopentenyl phosphate kinase (IPK) was improved for about sevenfold by rationally analyzing the coevolution of IPK protein sequences, so that the recombinant *E. coli* strain produced 97% more *β*-carotenoids than the starting strain [52]. Morita et al. demonstrated the synthesis of several unnatural polyketide-alkaloid scaffolds by exploiting a type III Polyketide synthase (PKS) using precursor-directed and structure-based approaches. The catalytic versatility of the type III PKS provides an excellent platform for further development of novel biocatalysts [53]. Computational, rational, or directed evolution engineering strategies can tailor a promiscuous enzyme for greater catalytic activity, thermostability or substrate specificity, and further increase the conversion efficiency from precursor to product.

### Transporter engineering

In microbes, excessive accumulation of plant secondary metabolites is usually toxic to the host, which can hamper cell growth and decrease PNP productivity. Thus, engineering transporters from both microorganisms and plants can improve the production of PNPs in microbial hosts [54, 55].

For example, overexpression of an efflux transporter from *Alcanivorax borkumensis* increased limonene production about 1.5-fold in engineered *E. coli* [56]. The native ABC transporter (ATP-Binding Cassette transporter) SNQ2 was overexpressed in the engineered *S. cerevisiae* that produced resveratrol, and the yield of resveratrol was increased from 48 to 61 mg/L after 48 h of fermentation [57]. Tripartite efflux pumps (pleiotropic resistant pumps) were constructed by combining TMDs (transmembrane domains) and NBDs (nucleotide binding domains) from endogenous transporters (AcrAB-TolC and MdtEF-TolC from *E. coli*) and heterologous transporters (MexAB-OprM from *Pseudomonas aeruginosa*), to improve isoprenoid production in *E. coli* [58]. The resulting chimeric transporter TolC-TolC-AcrB improved the specific yield of amorphadiene by 118% and kaurene by 104% [58]. Moreover, the co-overexpression of multiple transporters (*tolC* combined with *macAB*, *emrAB* and *emrKY*) enhanced the titer of amorphadiene more than threefold [59]. Recently, ginsenoside efflux pumps from *Panax ginseng* were identified as PDR (pleiotropic drug resistance homolog) transporter subfamily, which is used for the export of ginsenosides from microbial cell factories engineered for ginsenoside production [60, 61]. In addition to pumping out the product, transporters are also involved in substrate uptake in microbial hosts. Leonard et al. introduced *Rhizobium trifolii MatB* and *MatC* (encoding malonate synthetase and malonate carrier protein, respectively) into a recombinant *E. coli* strain, which introduced malonate uptake mechanism to increase synthesis of malonyl-CoA in flavonoid biosynthesis pathway [62].

With the broad and deep development of pathway identification and microbial metabolic engineering strategies, the accumulation and transportation of substrates, intermediates and products at the cellular/subcellular level has become more significant in cell factories, and has attracted growing attention. Accumulated knowledge about the function, structure and mechanism of transporters will facilitate the rearrangement of mass transport for the construction of more sophisticated cell machines in cell factories through transporter engineering.

## Engineered microbial cell factories for industrial-scale application

Several microbial cell factories have been improved through decades of efforts, reaching a level of productivity which is acceptable for industrial applications. The production of artemisinic acid, resveratrol and lycopene has increased by tens or hundreds of times, and has reached or is close to an industrial scale. Here, we summarize some successful industrialized cases of PNP production by transgenic microbial hosts to emphasize the viability and prospects of the commercial application of microbial cell factories.

### Artemisinin

Artemisinin is acknowledged as an effective pharmaceutical compound for the treatment of malaria [63], a serious disease that in 2015 alone affected 214 million people (African 88%, South-East Asia Region 10%), causing 438,000 deaths [64]. The demand for artemisinin is exponentially increasing every year because of the increased incidence of drug-resistant malaria throughout the world [65]. However, the concentration of artemisinin in the plant *Artemisia annua* is very low (0.01–1.1%), and improvement of the yield of artemisinin through plant breeding or total organic synthesis remains a challenge. Nevertheless, the synthesis or semi-synthesis of artemisinin using recombinant microorganisms is a promising solution. For more than 10 years, efforts have been made to improve the microbial production of precursors of artemisinin, and remarkable achievements have been made [39, 40, 66–69]. By engineering the genotype and carbon flux of *S. cerevisiae*, the yield of artemisinic acid reached 0.65 g/L in flask fermentation, starting from an initial 0.11 g/L [39]. When the recombinant strain was cultured in a well-controlled fermenter, and when the extractive solvent isopropyl myristate (IPM) was added, 25 g/L artemisinic acid was produced, which was about 35-fold higher than in the flask. Therefore, the production of artemisinic acid has been improved from 0.1 to 25 g/L in engineered *S. cerevisiae* through in vivo carbon flux rewiring and optimization of fermentation conditions [66], achieving the semi-synthesis of artemisinin (combined with one-step photochemical catalysis) at an industrial scale. Amyris Inc. has been engaged in pushing the semi-synthesis of artemisinin into commercial production.

### Resveratrol

Resveratrol is a polyphenolic compound found in several plant species, such as bush berries, peanuts, cranberries, and grapes. Resveratrol has been proved to decrease the risk of heart disease, diabetes and cancer (reviewed in [70]). It is widely used in medicine, as well as the health and cosmetic industries [71]. It is one of the fastest growing nutritional supplements in the flavonoid market [3]. According to a Frost & Sullivan report, in 2012 the global supply market value of resveratrol was about $50 million [72]. Due to the complexity and contamination problems in chemical synthesis of resveratrol, currently it is mostly extracted from plants [73]. Since the concentration of resveratrol in plants is extremely low, its production is limited and unsustainable. Recently, efforts have been focused on engineering microorganisms to synthesize resveratrol through fermentation [73]. Beekwilder et al. first introduced 4CL_2_ (4-coumaroyl-CoA ligase) from *Nicotiana tabacum* and STS (stilbene synthase) from *Vitis vinifera* into *E. coli*, and produced 16 mg/L resveratrol from 4-coumaric acid [74]. Many studies have also focused on metabolic engineering of *E. coli* cell factories towards resveratrol production [75–77]. Leam et al. reported an engineered *E. coli* with high resveratrol yield, which was constructed by using a stilbene synthase modification and enhanced intracellular malonyl-CoA supply, resulting in a final resveratrol titer of 2.3 g/L [78]. The budding yeast *S. cerevisiae* has also been engineered as a host for resveratrol synthesis. Durhuus et al. reported a recombinant *S. cerevisiae* strain producing about 5 g/L resveratrol [79], which is the highest resveratrol titer from microbial cell factories until now. The Evolva company has successfully accomplished industrial production of resveratrol in a yeast cell factory.

### Carotenoids

Carotenoids are a class of tetraterpenoids containing 40 carbons, with many important members such as lycopene, α-carotene, β-carotene, canthaxanthin, zeaxanthin, astaxanthin and lutein, which are used in various industries [80]. Especially, lycopene and astaxanthin are two of the most potent antioxidants among the dietary carotenoids, and may help lower the risk of chronic diseases including cancer and heart diseases [81–83]. The global market of carotenoids was $1.5 billion in 2014, and is expected to reach nearly $1.8 billion in 2019, with a compound annual growth rate (CAGR) of 3.9% [84]. In recent years, producing carotenoids through microbial fermentation has attracted intense attention [85]. Alper et al. reported that by using systematic (model-based) and combinatorial (transposon-based) methods to identify gene knockout targets, they obtained a maximum lycopene yield of 18 mg/g DCW (Dry Cell Weight), which represents an 8.5-fold increase over the recombinant *E. coli* K12 without the knockouts [86]. Cho et al. reported that the production of lycopene could reach as high as 1754 mg/L (38.1 mg/g DCW) in *E. coli* transformed with the *mvaK1*, *mvaD*, *mvaK2*, *mvaE*, *mvaS* and *idi* genes [87]. Although the engineered model microorganisms like *E. coli* and *S. cerevisiae* have not yet been successfully applied at the industrial scale, some microorganisms that endogenously produce carotenoids can be made applicable for industrial production through screening or mutation. Marcos et al. reported that a selected *Blakeslea trispora* strain produced 3.6 g/L lycopene in a dichloromethane-extracted fermentation [88]. Recently, lycopene and astaxanthin have been successfully produced commercially through fermentation of *Blakeslea trispora* [89] and *Phaffia rhodozyma* [90], respectively.

### Challenges in the fermentation process

Although developments in “omics” technology and synthetic biology have accelerated the pace of construction of microbial cell factories, it is still difficult to drive cell factories on an industrial scale. Only a few PNPs can be produced commercially so far. The essential problem in industrialization of microbial cell factories is how to cut down the production costs. In addition to the summarized strategies for optimizing microbial production in this context, there are still challenges in the field of fermentation process engineering. Two challenges we are facing right now are the engineering of microbial hosts toward the utilization of low-cost feedstocks, and improving microbial host resistance toward robust fermentation conditions.

Glucose is the most widely used material for the production of PNPs, including flavonoids, terpenoids, alkaloids and so on. However, glucose is not favorable in large-scale production because of the high price of raw materials and potential threats to food security. Therefore, engineering microbial hosts to use low-cost, non-food materials is extremely pertinent. Price et al. developed an engineering strategy to manipulate supramolecular enzyme assemblies which dramatically enhanced the carbon flux from methanol to the key intermediate fructose-6-phosphate in the microbial metabolic network, which provides a platform for biological conversion of methanol to higher value-added chemicals [91]. In 2016, Antonovsky et al. reported a non-native carbon fixation cycle that can synthesize sugars and other major biomass components from CO_2_ in *E. coli*, where all of the pathway intermediates and products are solely synthesized using CO_2_ as an inorganic carbon source and pyruvate as an energy source [92]. Although the engineering of microbial hosts to utilize low-cost materials is still not efficient, it will no doubt bring revolutionary progress to the entire cell factory industry, and therefore deserves further attention.

Since the 1930s, microorganisms have been used for industrial fermentation. *E. coli* and *S. cerevisiae* are two widely used hosts, as models for prokaryotic and eukaryotic expression systems, respectively. Recently, other microorganisms such as *Corynebacterium glutamicum* [93], *Streptomyces* spp. [94], *Yarrowia lipolytica* [80] and *Pichia pastoris* [95] have also been used as potential hosts for the production of PNPs. However, to achieve sufficiently high yields, the fermentation process of these microbial hosts requires precise control of factors such as temperature, pH, aeration, stirring, carbon source, nutritional supplements and antibiotics, resulting in high operating costs. Thus, engineering microbial hosts to be more competitive under robust fermentation conditions is another way to achieve cost saving. Shaw et al. engineered *E. coli, S. cerevisiae* and *Y. lipolytica* fermentations by supplying essential growth nutrients in the form of xenobiotic or ecologically rare chemicals, omitting the need for sterilization or antibiotics [96]. Yue et al. reported that a recombinant halophilic *Halomonas campaniensis* LS21 can produce the bioplastic PHB (polyhydroxybutyrate) in an energy-saving (non-sterilization), seawater-based, long-lasting and continuous open process, which largely saved fermentation costs [97]. This method provides microbial competitive advantages with minimal external risks, given that the engineered microbial hosts possess improved fitness within the customized fermentation environments.

## Conclusions

This review summarizes recent progress in the identification of novel biosynthetic pathways, engineering of microbial cell factories and industrial fermentation for the production of plant natural products (PNPs). Taking advantage of biological “omics” technologies and synthetic biology, more and more PNPs are being synthesized using microbial cell factories. Nevertheless, a huge number of PNPs remains to be investigated and overcoming current challenges will certainly open the doors for the valorization of a multitude of natural compounds supply through synthetic biology.
